# Deep learning‐based survival analysis for brain metastasis patients with the national cancer database

**DOI:** 10.1002/acm2.12995

**Published:** 2020-08-13

**Authors:** Noah Bice, Neil Kirby, Tyler Bahr, Karl Rasmussen, Daniel Saenz, Timothy Wagner, Niko Papanikolaou, Mohamad Fakhreddine

**Affiliations:** ^1^ Department of Radiological Sciences UT Health San Antonio San Antonio TX 78229 USA

**Keywords:** brain metastasis, deep learning, machine learning, survival

## Abstract

**Purpose:**

Prognostic indices such as the Brain Metastasis Graded Prognostic Assessment have been used in clinical settings to aid physicians and patients in determining an appropriate treatment regimen. These indices are derivative of traditional survival analysis techniques such as Cox proportional hazards (CPH) and recursive partitioning analysis (RPA). Previous studies have shown that by evaluating CPH risk with a nonlinear deep neural network, DeepSurv, patient survival can be modeled more accurately. In this work, we apply DeepSurv to a test case: breast cancer patients with brain metastases who have received stereotactic radiosurgery.

**Methods:**

Survival times, censorship status, and 27 covariates including age, staging information, and hormone receptor status were provided for 1673 patients by the NCDB. Monte Carlo cross‐validation with 50 samples of 1400 patients was used to train and validate the DeepSurv, CPH, and RPA models independently. DeepSurv was implemented with L2 regularization, batch normalization, dropout, Nesterov momentum, and learning rate decay. RPA was implemented as a random survival forest (RSF). Concordance indices of test sets of 140 patients were used for each sample to assess the generalizable predictive capacity of each model.

**Results:**

Following hyperparameter tuning, DeepSurv was trained at 32 min per sample on a 1.33 GHz quad‐core CPU. Test set concordance indices of 0.7488 ± 0.0049, 0.6251 ± 0.0047, and 0.7368 ± 0.0047, were found for DeepSurv, CPH, and RSF, respectively. A Tukey HSD test demonstrates a statistically significant difference between the mean concordance indices of the three models.

**Conclusion:**

Our results suggest that deep learning‐based survival prediction can outperform traditional models, specifically in a case where an accurate prognosis is highly clinically relevant. We recommend that where appropriate data are available, deep learning‐based prognostic indicators should be used to supplement classical statistics.

## 1 Introduction

### 1.A Clinical motivation

The median survival time for patients with brain metastases is on the order of months; however, some groups of patients can significantly outlive the median survival. Physicians have many treatment options to choose from, where the potential for disease‐free recovery is strongly connected to the treatment intensity. The brain met graded prognostic assessment (GPA) is one clinical tool that allows physicians to predict the longevity of patients with brain metastasis and thus select an appropriate treatment based on expected patient lifetime. For example, patients expected to live longer than 6 months are more likely to benefit from the short‐term memory protection offered by pin‐point radiosurgery treatment. On the other hand, patients with more limited life expectancy may be just as well served with a simpler whole brain radiotherapy, as they may not live long enough to experience the longer‐term cognitive effects of radiotherapy.

**Fig. 1:**
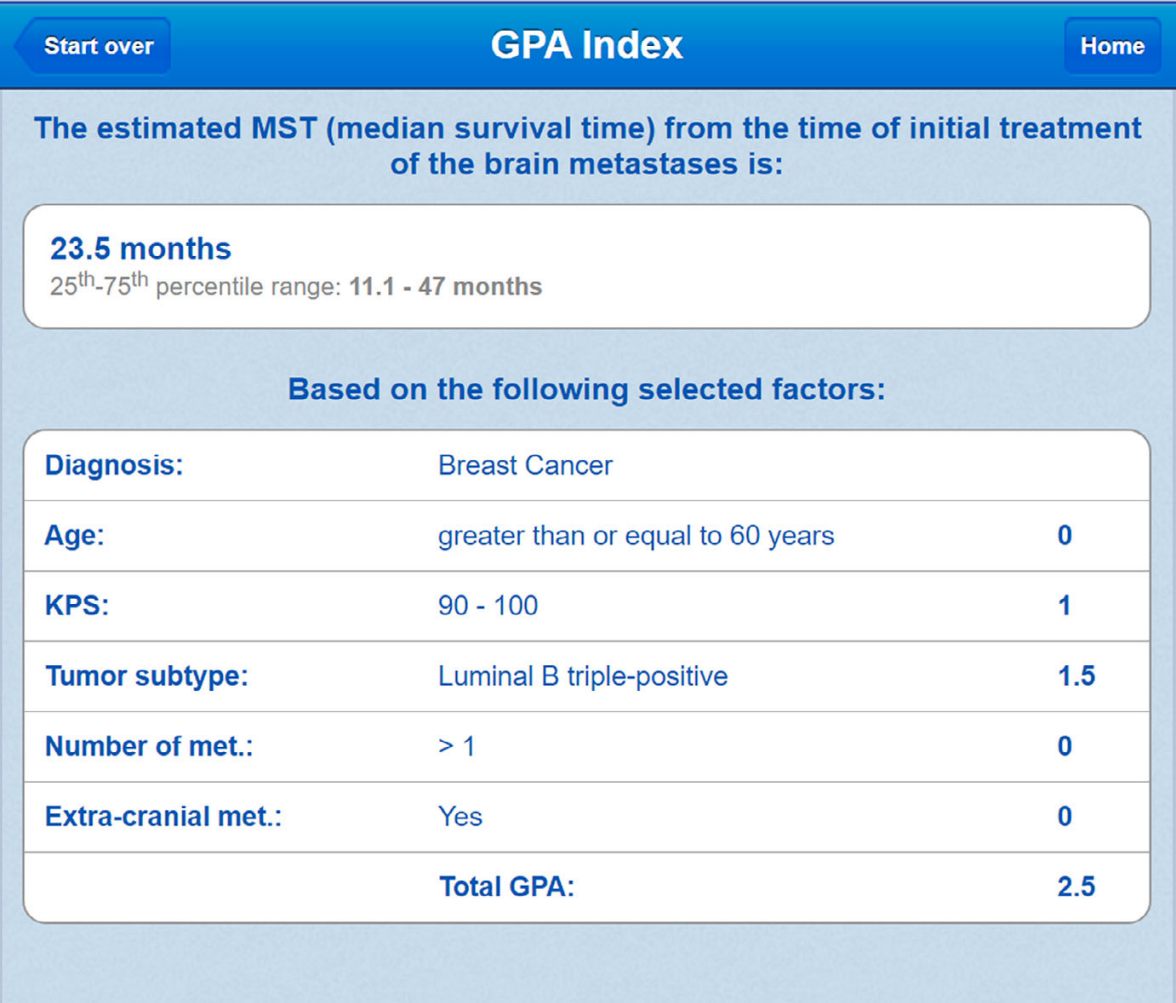
GPA is an online free‐to‐use tool utilized by some oncologists for patient prognosis (www.brainmetgpa.com). This validity of this tool has been established by Sperduto et al. in several journal publications with timely updates (reference: PMID: 22203767). The GPA tool uses five covariates determined to be significant by MCR to make survival predictions for patients with brain metastases and breast primary site. In this image, a breast cancer patient with age> 60, KPS in the range 90–100, tumor subtype luminal B triple‐positive, etc. is given a score of 2.5, indicating an expected survival of 11.1–47 months.

Brain met GPA uses multivariate Cox regression (MCR) and recursive partitioning analysis (RPA) to determine factors that significantly contribute to survival predictions. In the specific case of breast cancer, the factors determined to be most significant include Karnofsky performance status, number of metastases, and hormone receptor characterization. The weights according to MCR are used to compute an index, or scale from 0.0 to 4.0, that maximizes separation between survival curves between groups. New patients are placed in a group according to a few features and given a highly nonspecific survival estimate as in Fig. 1.^1^, ^2^

In this study, we focus on predicting survival probabilities for patients with brain metastases and breast primary site using a deep neural network. We expect that a representation learning approach to prognostic assessment will produce more accurate survival estimates than MCR and RPA for our dataset. Similar work in machine learning for patient prognosis has been done by Alcorn et al.^3^ Their work focuses on the application of random survival forests specifically to the problem of prognosis for patients with bone metastases.

### 1.B Cox proportional hazards

Proportional hazards models are regarded as the gold standard for survival analysis.^4^ Cox models aim to describe a patient‐specific hazard function (event rate), given a quantitative description of their attributes (covariates, features).^5^ According to the proportional hazards assumption, the event rate for patient having covariates x at time t is modeled with the hypothesis function.

$$h\left(t|x;\Theta \right)=h\left(t\right)e^{\Theta^{T}x};\;x,\;\Theta \in R^{n}.$$

Regression with survival data is limited by censoring, or “loss to follow‐up.” There is no meaningful way to ascribe an event time to patients who discontinue communication with record keepers. Therefore, the parameters of the Cox model must be learned with a nonparametric objective function. Parameters $\Theta^{*}$ that best predict the order of survival times for *N* patients having covariates {$x^{\left(1\right)},\ldots ,x^{\left(N\right)}\}$ and survival times $\left\{t^{\left(1\right)},\ldots ,t^{\left(N\right)}\right\}$ are obtained by maximizing the Cox partial likelihood:

$$\Theta^{*}=\begin{array}{c} \text{argmax}\\ \Theta \end{array}\prod_{i:\delta_{i=1}}\frac{h\left(t^{\left(i\right)}|x^{\left(i\right)};\Theta \right)}{\sum_{j:t^{\left(j\right)}>t\left(i\right)}h\left(t^{\left(i\right)}|x^{\left(j\right)};\Theta \right)},$$

where $\delta_{i}=1$ indicates that patient *i* was not lost to follow‐up.

### 1.C DeepSurv

Deep learning has been shown to be an effective tool for modeling nonlinear functions. There have been many breakthroughs in image classification, natural language processing, and other fields due to new methods and increased availability of deep learning platforms.^6^ In 2016, Katzman et al. released DeepSurv, a Cox deep neural network for public use. DeepSurv assumes the same structure as the Cox proportional hazards (CPH) model but uses a state‐of‐the‐art neural network to evaluate risk ($\Theta^{T}x$ in CPH).^7^ The deep architecture of the model allows it to create higher‐order representations of features that might be more useful for survival predictions than the features alone. DeepSurv has been used to successfully characterize risk in several datasets including the Worcester Heart Attack Study (WHAS) and Molecular Taxonomy of Breast Cancer International Consortium (METABRIC) datasets. DeepSurv realizes its potential fully when the true risk function is highly nonlinear. DeepSurv’s concordance index improved from CPH by about 15% on toy data with risk generated from a 2D Gaussian.

### 1.D Random survival forests

For comparison of DeepSurv with a leading nonlinear machine learning method that is not based on deep learning, a random survival forest was implemented in R using the RandomForestSRC package. Random Survival Forests utilize an ensemble of trees generated from bootstrap samples of the training dataset. Trees are “grown” for each data subset by identifying the covariate from a random set of candidate covariates that maximizes the survival difference for the data subset. The surviving fraction of the split data is calculated for each terminal node in the decision tree. For prediction on a test data point, the data are passed through all trees and the ensemble cumulative hazard is used to make survival predictions.

## 2 Methods

### 2.A NCDB dataset

Survival predictions of three machine learning models were compared using 1673 patients’ data from the National Cancer Database. Patients included in the dataset all had brain metastases and a breast primary site. Twenty‐seven features including age, staging information, and hormone receptor status were used to characterize risk. An event indicator and time was provided for each patient.

**Fig. 2:**
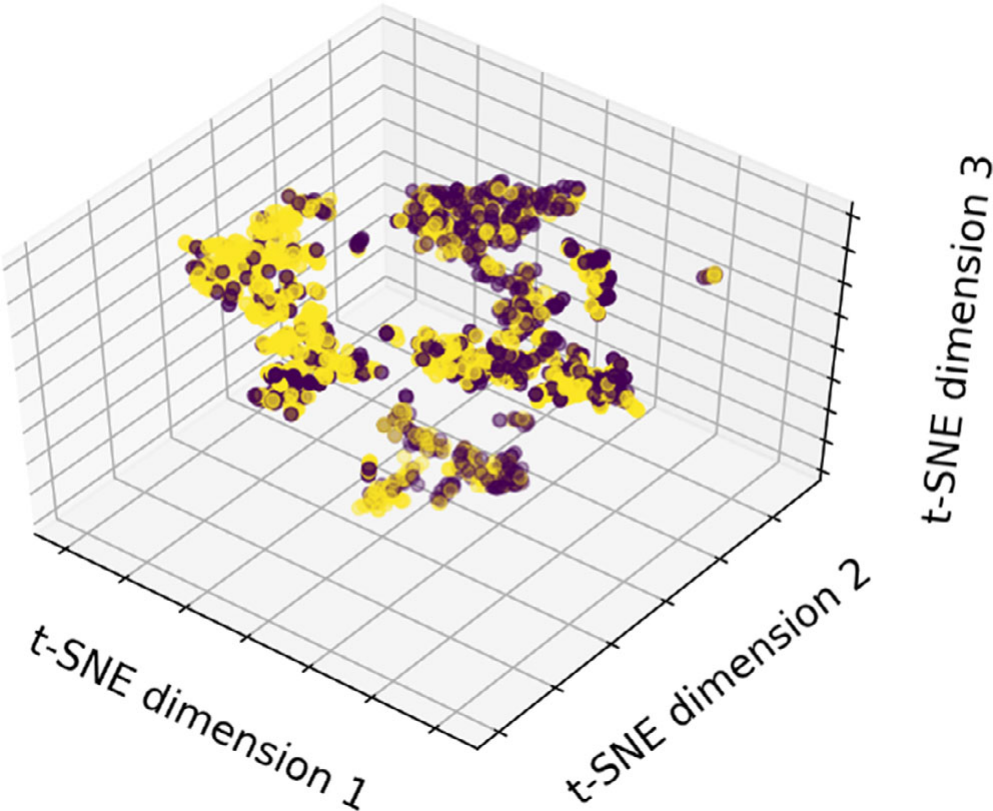
t‐SNE visualization of the NCDB dataset, labeled by event occurrence. Patients with recorded death times have a yellow marker, while those which are lost to follow‐up are labeled purple. Because directions in the embedding space do not correspond to known physical parameters, axis labels in t‐SNE visualization are arbitrary.

The NCDB dataset can be visualized with dimensionality reduction. t‐Distributed Stochastic Neighbor Embedding is a method of dimensionality reduction that compromises large distances in data space in favor of preserving small distances.^8^ A three‐dimensional embedding of the 27 features was created using t‐SNE in Python (Fig. 2). Clustering by event occurrence suggests that there are learnable representations of the dataset that describe patient survival.

Monte Carlo cross‐validation with 50 samples (84% training, 16% test) was used to assess model performance.^9^ The three models considered were DeepSurv, CPH, and a random survival forest (RSF). The RSF serves as a benchmark nondeep state‐of‐the‐art survival analysis method, based on recursive partitioning analysis. For more on RSFs, see.^10^, ^11^

### 2.B Survival model evaluation

The generalization error for a model with parameters Θ can be described by its concordance index $\hat{C}$ on a test data set,^12^, ^13^

$$\hat{C}=\frac{1}{N}\sum_{i:\delta_{i}=1}\sum_{j:t^{\left(i\right)}<t^{\left(j\right)}}1\left[\Theta^{T}x^{\left(i\right)}>\Theta^{T}x^{\left(j\right)}\right].$$

The concordance index considers all admissible pairs of patients and computes the fraction of patients that are correctly ordered by the model according to their true survival times. A pair of patients $\left(A,B\right)$ is considered admissible if patient *A* has survival time $t_{A}<t_{B}$ and experiences an event, $\delta_{A}=1$. Concordance indices range from 0.0 (100% discordant) to 1.0 (100% concordant), with $\hat{C}=0.5$ suggesting that predictions were made randomly.

### 2.C Model implementation

Cox regression was implemented in Python with stochastic gradient ascent. Parameters were updated according to their derivatives with respect to the Cox partial log‐likelihood:

$$\Theta_{k}:=\Theta_{k}+\alpha \sum_{i:\delta_{i}=1}\left(x_{k}^{\left(i\right)}‐\frac{\sum_{j:t_{j}\geq t_{i}}x_{k}^{\left(j\right)}e^{\Theta^{T}x^{\left(j\right)}}}{\sum_{j:t_{j}\geq t_{i}}e^{\Theta^{T}x^{\left(j\right)}}}\right),$$

where α is the learning rate. Updates were halted when the validation concordance appeared to converge to a maximum (60 iterations). A Wald test with significance level α = 0.01 was used to identify the parameters which are likely to be truly nonzero in the Cox framework.^14^ Features and significance levels are displayed in Table 1.

**Table 1:** The significance of various covariates according to the proportional hazards assumption is listed in the table.

Variable	*P*	Variable	*P*	Variable	*P*
Age	0.02	Sex	0.64	Race	0.51
Charleon/Deyo	0.19	**Grade**	**0.01**	Tumor Size	0.87
Regional LNs Positive	0.94	**AJCC Clinical N**	**<0.005**	AJCC Pathologic N	0.32
Bone Mets at DX	0.59	**Brain Mets at DX**	**<0.005**	Lung Mets at DX	0.85
ER Assay	0.45	PR Assay	0.93	**HER2 Summary**	**<0.005**
Multigene Signature Method	0.62	Multigene Signature Results	0.91	**Treatment Days After DX**	**0.01**
Rad Days After DX	0.38	Radiation Type	0.97	**Volume Irradiated**	**<0.005**
Regional Dose	0.99	Chemotherapy Type	0.16	Hormone Therapy Type	0.26
**Immunotherapy Type**	**<0.005**	Mets at DX	0.42	Systemic Surgery Sequence	0.04

Note

Some factors that are obviously significant in survival predictions have significant *P*‐values. The many covariates with insignificant *P*‐values will not greatly contribute to risk calculation in the CPH framework. Bold indicates statistical significance.

**Fig. 3:**
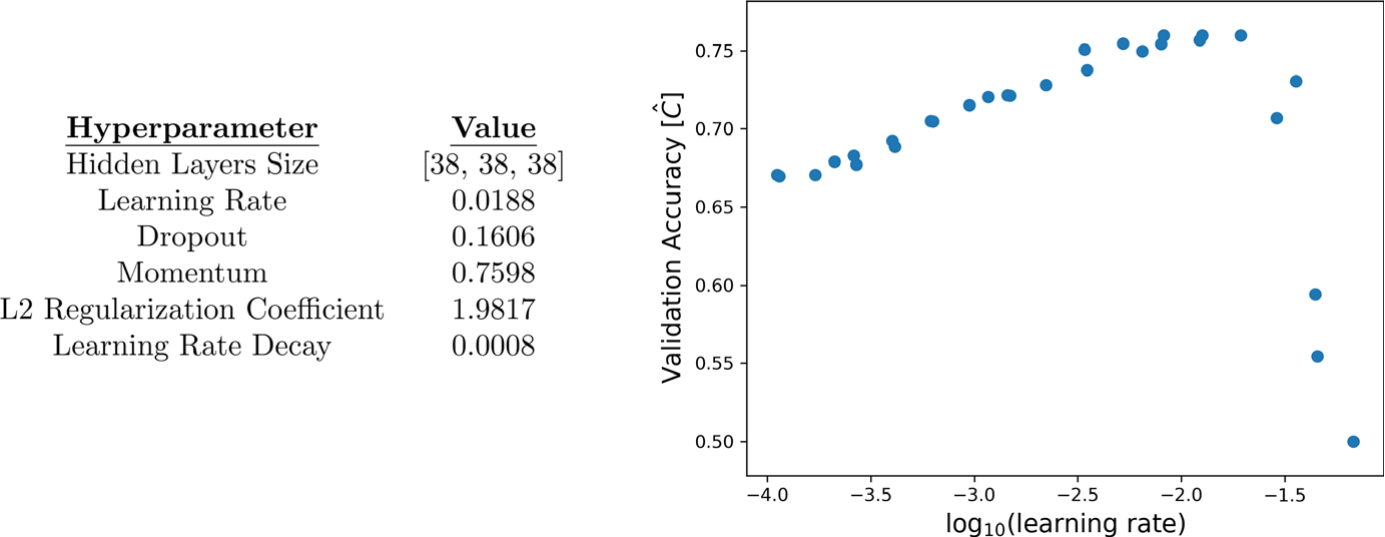
*Left:* Hyperparameters that yielded the highest validation accuracy. *Right:* A plot of learning rates versus validation accuracy demonstrates the impact of one hyperparameter choice on generalization error. A learning rate of $10^{‐1.726}=0.0188$ was chosen for deployment.

The deep neural network DeepSurv was implemented in Python with L2 regularization, batch normalization, dropout, Nesterov momentum, and learning rate decay. A six‐dimensional box in hyperparameter space was uniformly sampled 100 times and DeepSurv’s performance was evaluated with a validation dataset.^15^ The hyperparameters that yielded the highest validation accuracy (Fig. 3) were chosen for deployment. DeepSurv was then trained for 7000 epochs per sample at about 32 min per sample on a 1.33 GHz quad‐core CPU.

## 3 Results

**Fig. 4:**
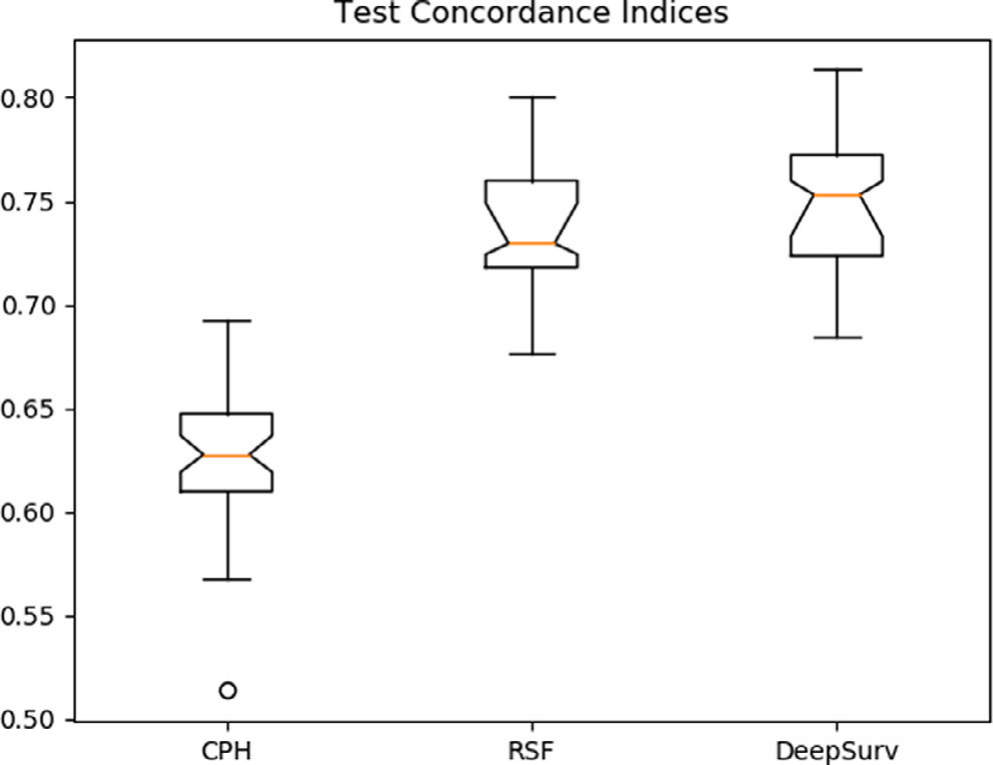
The test set concordance indices for three models are shown in this plot. The 95% confidence intervals of the median are given by notches in the boxes. DeepSurv appears to yield the smallest generalization error.

The three models were independently trained and validated 50 times on the randomly split dataset. Test set concordance indices of ${\hat{C}}_{\mathrm{Deep}}=0.7488\pm 0.0049$, ${\hat{C}}_{\mathrm{CPH}}=0.6251\pm 0.0047$, and ${\hat{C}}_{\mathrm{RSF}}=0.7368\pm 0.0047$, were found for each model. DeepSurv and the RSF significantly outperform the CPH model. Concordance indices are displayed in Fig. 4.

**Fig. 5:**
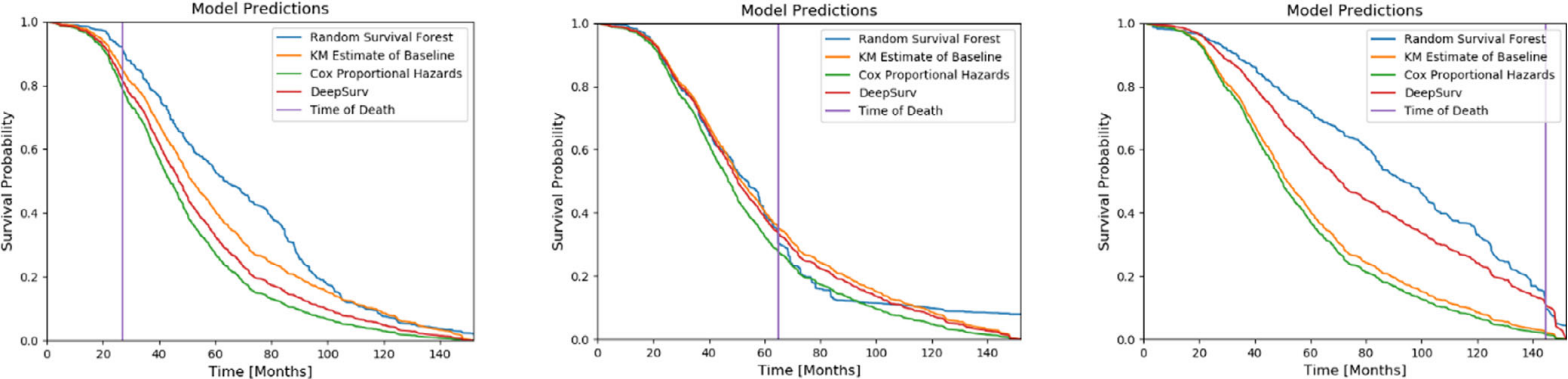
Survival probability curves $S\left(t\right)=p\left(T>t\right)$ for three patients predicted by each model according to a Kaplan–Meier estimate of the baseline survival. The true times of death for these patients are indicated by the vertical lines.

A Tukey Honestly Significant Difference test was used to evaluate the difference of mean concordances for each model (Table 2). With a significance level of 0.05, one can reject the null hypothesis that the mean concordances for DeepSurv and the RSF equal the mean concordance for the proportional hazards model. There is not enough evidence to suggest that the mean concordance of DeepSurv is different from the mean concordance of the random forest Fig. 5.

**Table 2:** A Tukey HSD post‐hoc test supports the hypothesis that DeepSurv and the RSF have greater mean test concordances than CPH.

Tukey HSD – Multiple comparison of means					
Group 1	Group 2	Difference of means	Lower bound	Upper bound	Reject null
DeepSurv	CPH	0.1237	0.1074	0.1399	True
RSF	CPH	0.1117	0.0957	0.1277	True
DeepSurv	RSF	0.0120	‐0.0042	0.0282	False

To highlight the clinical utility of working survival models, we have included predicted survival curves for three test patients in one cross‐validation sample. The CPH and DeepSurv predictions are calculated by exponentiating a Kaplan–Meier estimate of the baseline survival function (from the training set) with the predicted hazard. The survival forest instead creates a unique curve based on all the terminal nodes to which the patient belongs.

**Fig. 6:**
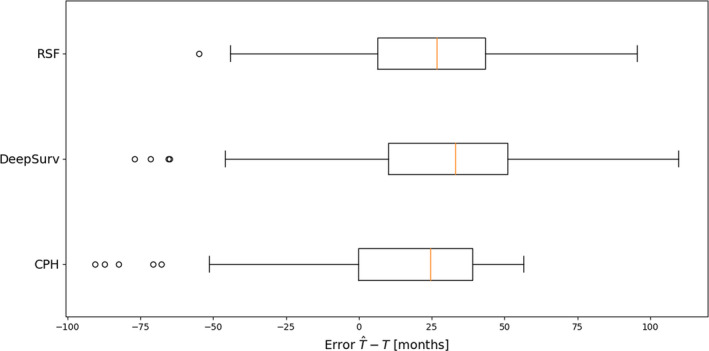
The distribution of errors predicted with $\int_{0}^{\infty }S\left(t\right)dt‐T$ for the validation dataset suggests all three models overestimate patient survival times.

With the assumption of a baseline survival function, the expected value $\hat{T}=E\left[T\right]$ for each patient in the validation dataset can be evaluated with $\hat{T}=\int_{0}^{\infty }S\left(t\right)dt$.^16^ This technique can be used to better understand the biases of the working models. We consider the distribution of errors $\hat{T}‐T$ for each model (Fig. 6). All three techniques tend to overestimate the true survival time.

## 4 Discussion/Conclusion

In this study we demonstrate a highly clinically relevant scenario where deep learning‐based survival estimates outperform the gold‐standard survival analysis technique — MCR. With an appropriate baseline hazard estimate, survival predictions generated by DeepSurv might prove beneficial to patients and physicians in determining an appropriate treatment option.

One failure of deep learning‐based survival analysis is the challenge of interpretation. Neural networks utilize complex interactions between features, which improves classification performance at the cost of interpretability. This is one weakness of deep learning compared to conventional methods. The interpretability of the inner workings of models in artificial intelligence is active area of research beyond the scope of this work.^17^, ^18^ We therefore recommend the supplement of traditional techniques with deep learning, rather than outright replacement. If future work allows intuitive interpretability of the “black box” of neural networks, they may well replace current models such as CPH.

Advanced survival analysis techniques suffer from inaccessibility. Brain met GPA is widely popular because it is online and easy to use. Any future deep learning‐based approaches to patient prognosis should be accessible to physicians in the form of a webpage or easy‐to‐use software.

One weakness of the deep risk framework is the lack of a time‐dependent hazard estimation; DeepSurv acts as an extension of the classic CPH model. Luck et al. have recently shown that by directly modeling the survival function, as opposed to risk, they can obtain concordance indices on par with those generated by DeepSurv.^19^ A final implementation of this work for clinical use might benefit from an effort to include time dependence.

Currently, there does not appear to be any significant benefit to using DeepSurv over the Random Forest. However, deep learning is a very rapidly growing field. DeepSurv, despite utilizing several state‐of‐the‐art training techniques (dropout, batch normalization, L2 regularization), is architecturally quite simple. It does not take advantage of expected patterns in survival data in the way that convolutional networks handle images, and recurrent networks handle language. Our group expects that deep learning‐based methods will continue to improve in the near future.

## CONFLICT OF INTEREST

The authors declare no conflict of interest.
